# Supplementary figures and images for: Nepalese version of Douleur Neuropathique 4 (DN4) questionnaire for detection of neuropathic pain signs and symptoms: Translation and psychometric properties
Source: PLoS One. 2023 Jul 17;18(7):e0287737. doi: 10.1371/journal.pone.0287737 (PMC10351728; doi:10.1371/journal.pone.0287737)

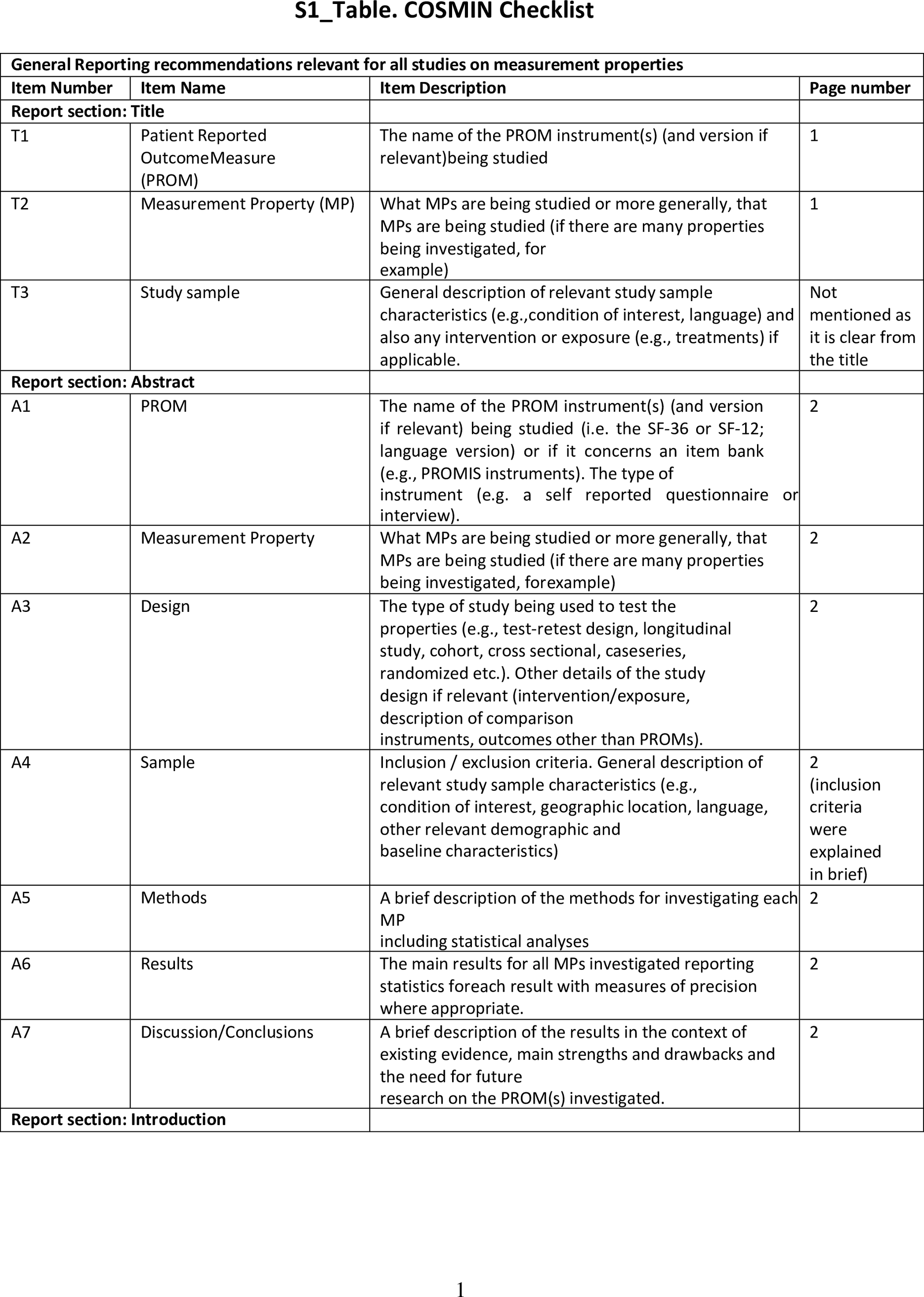

Supplement: S1 Fig — (TIF) [file pone.0287737.s001.tif]

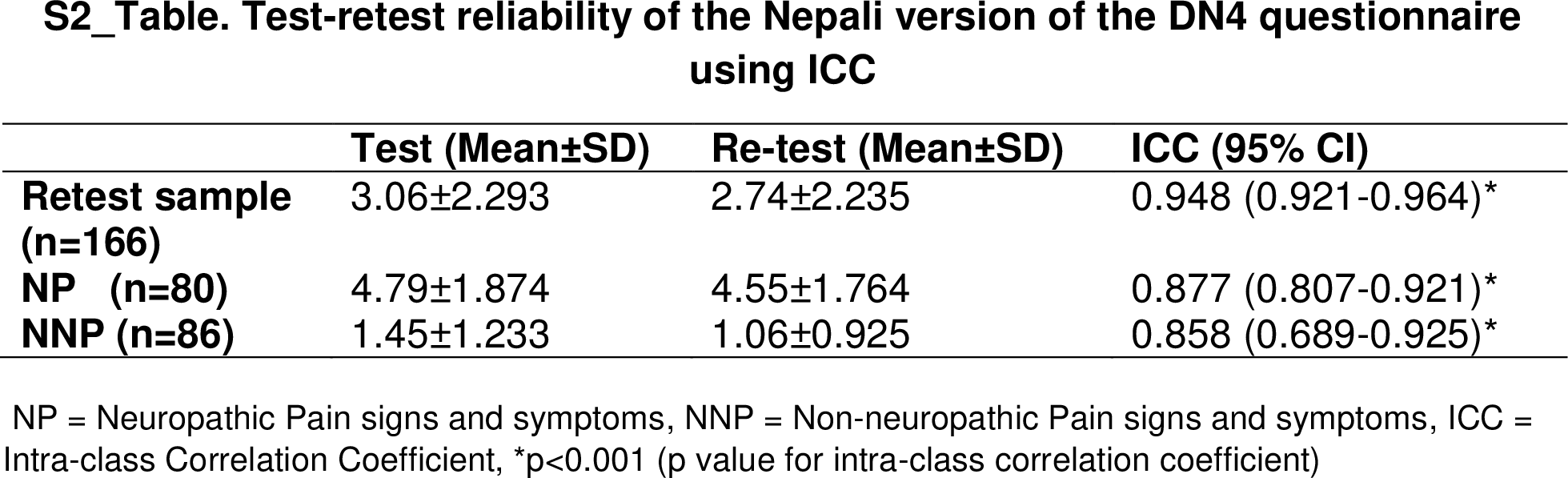

Supplement: S2 Fig — (TIF) [file pone.0287737.s002.tif]

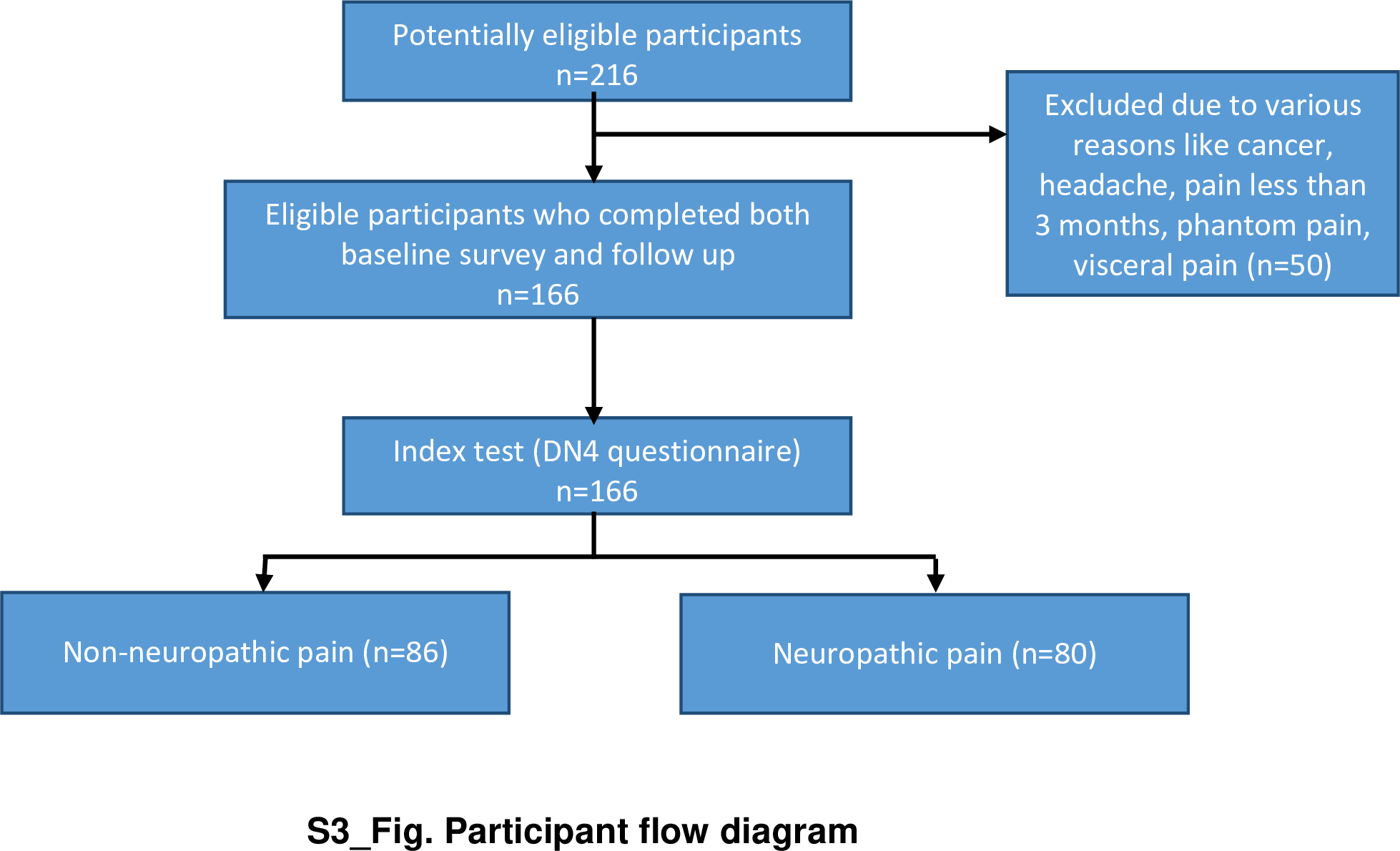

Supplement: S3 Fig — (TIF) [file pone.0287737.s003.tif]

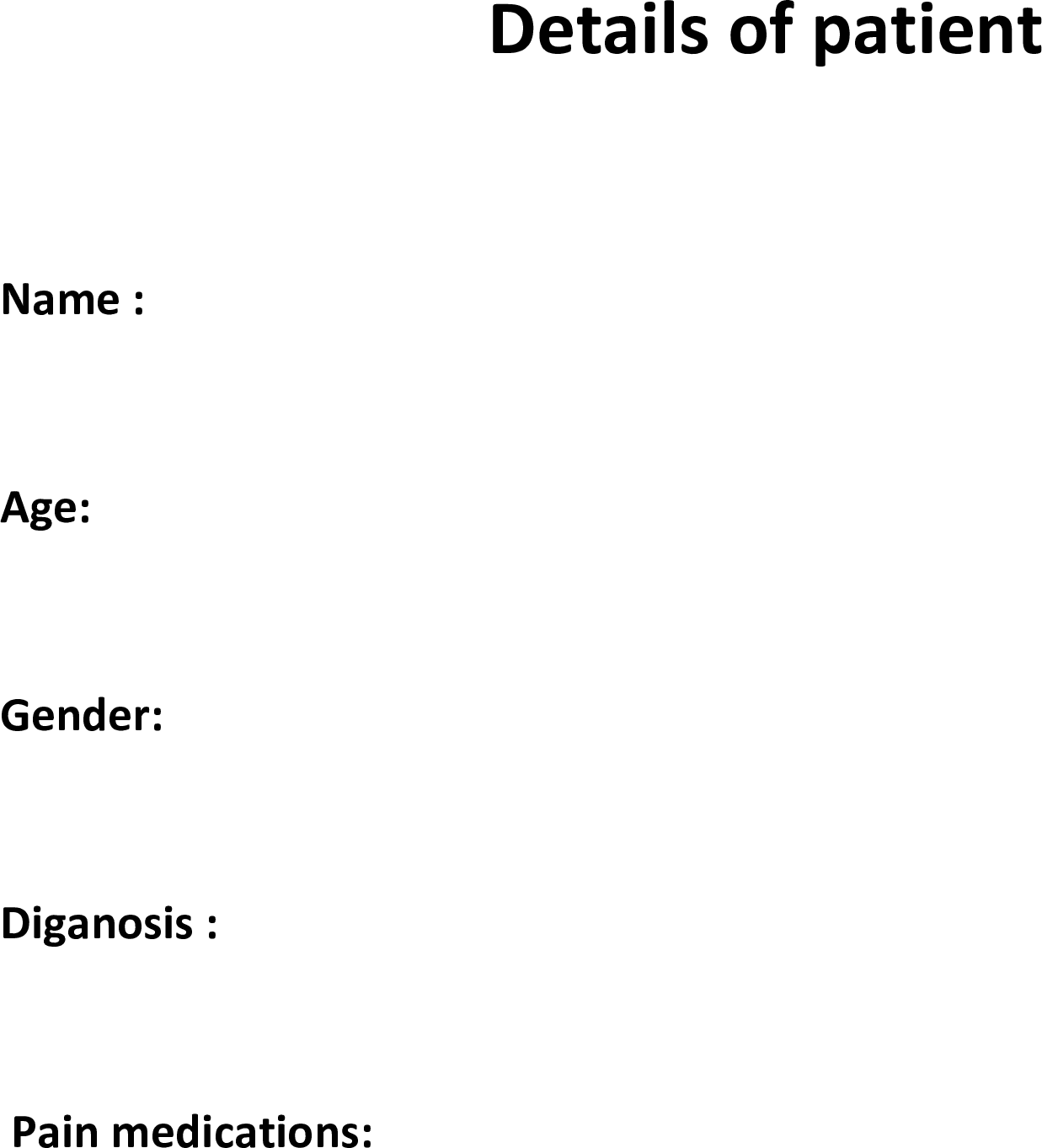

Supplement: S4 Fig — (TIF) [file pone.0287737.s004.tif]

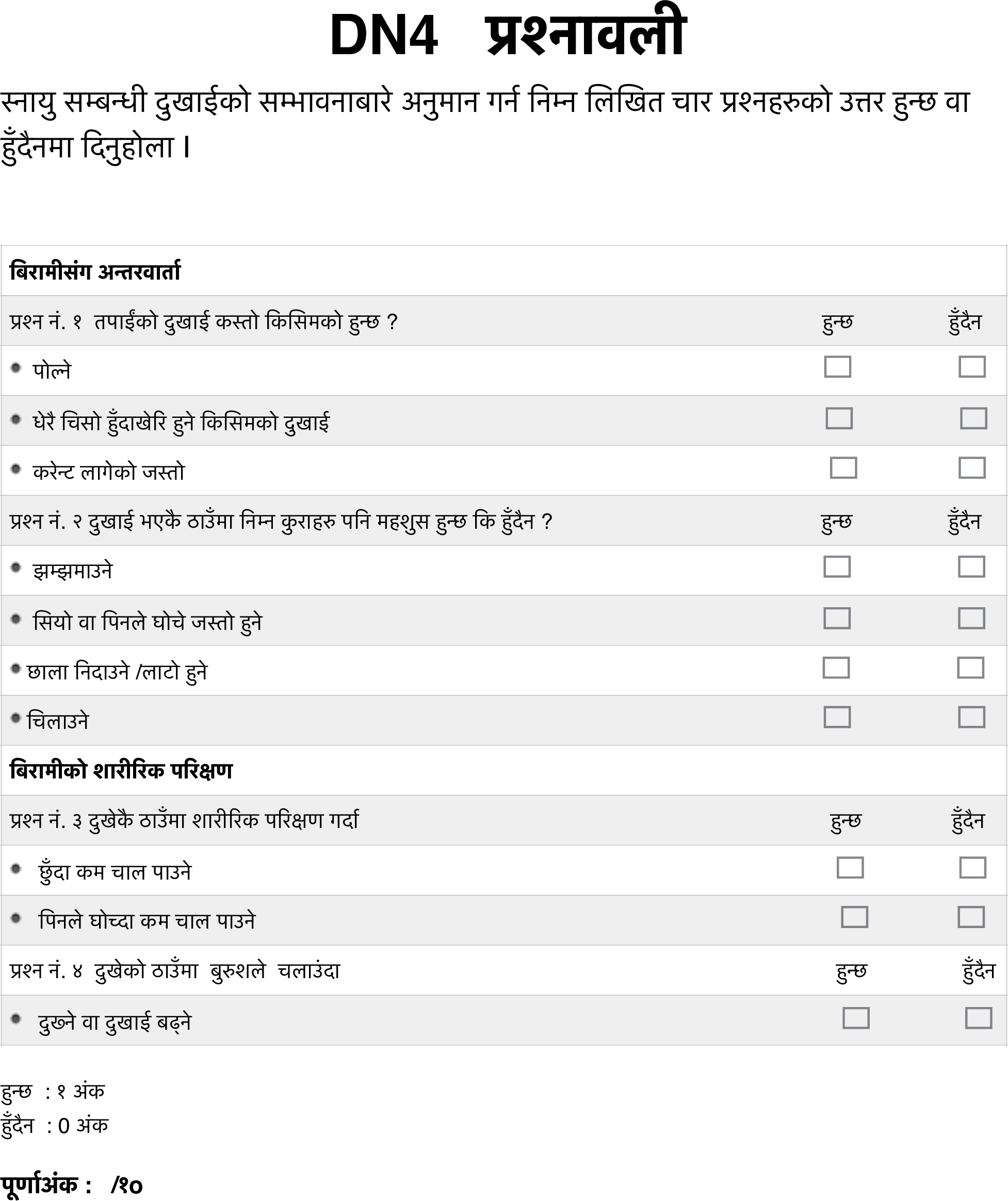

Supplement: S5 Fig — (TIF) [file pone.0287737.s005.tif]
