# Supplementary material for: Nepalese version of Douleur Neuropathique 4 (DN4) questionnaire for detection of neuropathic pain signs and symptoms: Translation and psychometric properties
Source: PLoS One. 2023 Jul 17;18(7):e0287737. doi: 10.1371/journal.pone.0287737 (PMC10351728; doi:10.1371/journal.pone.0287737)
Supplement: Attachment — Submitted filename: Response to Reviewers.docx [file pone.0287737.s007.docx]

To

Dr. Steve Zimmerman

Associate Editor, PLOS ONE

Dear Dr. Steve Zimmerman,

**RE: PONE-D-21-36866 - “Nepalese version of Douleur Neuropathique 4 (DN4) questionnaire for assessment of neuropathic pain: a validation study”**

Thank you so much for your email received on 12^st^ August 2022 and the valuable suggestions of the reviewers. We have attempted to address all these comments and give a point-by-point response.

Please find attached the following for your kind consideration:

1) A rebuttal letter that responds to each comment of the reviewers
2) A marked-up copy of the manuscript highlighting changes as per the reviewers’ comments.
3) A clean version of the revised manuscript without track changes

Hope the changes made have addressed all your comments and suggestions. We are always available for any further information if required.

Yours Sincerely,

Sujata Shakya

Corresponding Author

**POINT BY POINT RESPONSES TO THE COMMENTS OF THE EDITOR AND REVIEWERS**

**PONE-D-21-36866**

**Response to the reviewers**

- Response: We thank the reviewers for reviewing the manuscript and very useful and encouraging comments. We have revised the manuscript in line with your suggestions. The specific changes and responses to the different points raised are mentioned below. Your comments are highlighted in bold followed by our responses.

**Response to reviewer 1**

**The assessment of neuropathic pain is a trend topic in the current research literature and may be a main focus of interest for readers.**

**This is a well-written manuscript with an important clinical message, and should be of great interest to the journal readers. However, from my point of view, authors should include the following requeriments**

**Introduction may be improved adding new information in order to provide an adequate state-of-the-art including some references. I suggest to include this references include in the attached to complete this requirement related to neuropathic Diabetic foot complications that authors do not included**

**DOI 10.1111/iwj.13263**

- Response: Thank you for the valuable comments and suggestion. As suggested by the Associate Editor of the journal that it is not essential to cite the papers on foot pain, we have not included the mentioned references in the introduction. However, we have added few references as suggested by reviewer 2, which we have mentioned under the response to reviewer 2.

**Methods are well-designed with relevant and complete information. Correct sample size calculations, good description of the properties of the outcome measurements as well as detailed statistical analyses were included.**

**Discussion section is well structured with different sections. Authors manage well the discussion leading a good comparison with the showed references.**

**However, author should discuss their results with regards other prior studies in validation and transcultural adaptation I suggest to include these references to attend this requeriment**

**doi:10.3390/ijerph17176141**

**DOI: 10.18632/aging.202140**

- Response: Thanks for your suggestion. We went through the mentioned literature. The first one was observational study and the second one was on validation of Edmonton Frail Scale (EFS), which is used to predict frailty disability outcomes among elderly. This tool is entirely different from the tool that we studied, which was meant to identify the neuropathic signs and symptoms. Therefore, as suggested by the Associate Editor, we have not included the mentioned references to compare our findings.

**Response to reviewer 2**

**Major criticisms:**

**1. The Introduction does not exactly cover why the study is needed. I would suggest the authors to include what is known about the topic in relation to Nepal, what the current gap is and how this paper addresses this major literature gap. You will see specific suggestions below.**

- Response: We have revised the introduction section mentioning the need for the validation of the DN4 questionnaire and the current status about the literature on neuropathic pain, which is lacking in our country. We have included recently published review article by Sharma et. al (2019) about the state of clinical pain research in Nepal.

**2. Methods: Critical steps and items of validation studies are not covered. I would recommend authors to use COSMIN Checklist to report the current paper.** [**https://www.cosmin.nl/wp-content/uploads/COSMIN-study-designing-checklist_final.pdf**](https://www.cosmin.nl/wp-content/uploads/COSMIN-study-designing-checklist_final.pdf)

- Response: We totally agree with the reviewer and have referred to the COSMIN checklist to report the methods.

**3. Results: Also use COSMIN checklist to report results.**

- Response: Thank you for the suggestion. We have checked the results with the COSMIN checklist. We have also attached the checklist in the separate file.

**4. Discussion: It is quite vague and non-focused. I would recommend authors to rewrite the Discussion section to keep it more focused. Please focus on what key results were, whey they mean, how are the similar/different from other studies in the same country/region (or international literature if local/regional research is lacking), what are the implications, limitations, recommendations for the future studies. To write more focused Discussion section, authors may refer to a BMJ editorial,** [**https://www.bmj.com/content/318/7193/1224**](https://www.bmj.com/content/318/7193/1224)

- Response: We have divided the discussion into three parts, the first involved the difficulties we faced during the translation process. In the second part, we compared our findings with those of other literature. We have added few more references for discussing and comparing our findings. Likewise, in the third part, we have stated the study limitations, implications for future studies and conclusion.

**5. The authors state at the end of introduction "Once validated, this tool can be used as a screening tool by any health personnel for the diagnosis of neuropathic pain." In order to facilitate this more widely, I strongly recommend authors to upload Nepali DN4 as either appendix or supplementary online file to promote wide use of the scale in clinical practice or research. This aligns quite closely with ethos of PLOS ONE. I also recommend authors to share their data openly.**

- Response: We have already uploaded Nepali DN4 tool as the supplementary file during online submission of our manuscript. You can check it under our submission portal.

**6. The authors indicate that DN4 is used to "diagnose neuropathic pain". Rather it is to identify signs and symptoms related to neuropathic pain. This is an important limitation of a self-reported measure and I request authors to change this consistently throughout the manuscript.**

- Response: We highly appreciate and agree with this comment by the reviewer. As suggested, we have revised accordingly throughout the manuscript.

**Specific comments:**

**INTRODUCTION:**

**1. Information presented in the Introduction is quite generic. I think authors should be more specific. Rather than only saying neuropathic pain is one of the prevalent conditions, also say what the prevalence is (Line 72); be specific on what other advanced investigations are and with citation please (line 71).**

- Response: We thank the reviewer for the comment. We have tried to make the introduction more specific by mentioning the exact prevalence of the neuropathic pain. We also listed what other advanced investigations are.

**2. As above, Line 76: Rather than detection of neuropathic pain, it is the identification of the "neuropathic signs and symptoms".**

- Response: We totally agree with the reviewer. We have now changed the sentence as per the suggestion.

**3. When authors indicated the previous translation of pain related instruments in Nepali, I was hoping to see their use in the current study to assess construct validity of the scale, but this was lacking. It is the strength that authors have mentioned prior translation of numerical pain rating scale in Nepali. But what is missing is "what is known about Neuropathic pain" in Nepal and availability of other screening tools in Nepali. On a quick PubMed search, self-reported LANSS has been translated and validated in Nepali (https://pubmed.ncbi.nlm.nih.gov/34583020/). The Introduction would benefit by such review of literature and to highlight any advantages of availability of DN-4 in addition to self-reported LANSS and other instruments.**

- Response: Thank you for the comment. The validation of Nepali Version of S-LANSS for assessment of neuropathic signs and symptoms was published only in March, 2022 by Saurav Sharma et. al. This was after completion of our data collection. Therefore, we did not find any other validated Nepali tool to include in our research.

**METHODS:**

**4. Could the authors add the name of the tertiary care hospital.**

- Response: Thanks a lot for the suggestion. We have now mentioned the name of the tertiary care hospital as ‘Tribhuvan University Teaching Hospital’.

**5. Line 105: I would suggest to use simple term such as "Nepali" than to refer to the language by "Nepalese language".**

- Response: As suggested by the reviewer, we changed the word ‘Nepalese language’ to ‘Nepali’.

**6. Line 106: Authors have provided comprehensive list of conditions which were excluded. It is unclear why these were excluded. Additionally, it is also important to know what are the types of chronic pain conditions that were included.**

- Response: We totally agree with the reviewer’s comment. We specifically mentioned what types of chronic pain conditions were included and what were the reasons for the exclusion.

**7. Line 123: Info about translation guideline belongs to the next paragraph on Phase 1: Translation and back translation.**

- Response: As suggested by the reviewer, we moved this line under phase 1.

**8. It is surprising to see 6 forward translations of the scale, I am unclear of the rationale why. It seems like rather waste of Human Resources. The paper authors cited does seem to support this.**

- Response: Thank you for the comments. I would like to make it clear that the forward translation of the scale was performed by only three individuals not 6, which is clearly mentioned in phase 1. Among the three, two were linguistic experts and one was neurologist.

**9: Please provide some info on why 25 patients were recruited for pre-testing.**

- Response: We have mentioned in Line 158 the reason for recruiting 25 patients for pre-testing.

**10. Line 148: State in how many people was the translation of "cold pain" was tested.**

- Response: Thank you for the comment. We have now clearly specified the total number of participants who were tested for cold pain.

**11. Line 150: It was odd that content validity of the scale was discussed after pre-testing. Ideally it is performed before pre-testing. Only upon all experts agree on the prefinal version, it is subjected to pretesting.**

- Response: We totally agree with the reviewer on this comment. The content validity of the scale was assessed before pre-testing, which was mistakenly mentioned after pre-testing in the earlier version of the manuscript. Now, it has be corrected.

**12. Test methods: Please explicitly state what was the diagnostic criteria based on the IASP used in the current study. This is important for the internal validity of the study.**

- Response: In the study limitation, we have mentioned that the diagnosis of neuropathic pain by the pain experts based on history taking and clinical examination was taken as standard, which is considered to be the probable neuropathic pain as per IASP guidelines. For the diagnosis of definitive neuropathic pain, advanced tests have to be used, which was not possible in our case due to financial constraints. So, we have revised our statement in the manuscript (Line 181-182)

**13. specify how test-retest reliability was assessed (agreement versus consistency)? Also specify Fleiss notation system (Shrout & Fleiss, 1979), which describes the type of ICC for example as ICC(2,1).**

- Response: We have mentioned both in the methods and results section that the test-retest reliability was assessed in two episodes in 2 weeks period. As per the Fleiss notation system, we used type C intraclass correlation coefficients, that is, ICC (3,1) which is a two-way mixed effects, consistency, single rater measurement. This was clearly explained in line 222-223.

**14. Line 178: Please describe the method how ROC curve was plotted and how AUC was derived. State what the scores of AUC mean.**

- Response: As per the suggestion of the reviewer, we have explicitly explained how ROC curve was plotted and how AUC was derived and interpreted in line 201-205.

**15. Line 180: Explain what each of the parameters mean (sensitivity, specificity and liklihood ratios) with explanation on how these were computed. State what these scores mean.**

- Response: All the parameters and their interpretations are explained in the methods section in line 209-217

**RESULTS:**

**16: The result would benefit with a flow diagram. How many patients were approached, how many recruited (and excluded), how many completed baseline survey, how many followed up at two weeks.**

- Response: We had shown the patient flow diagram as a supplementary file, where we presented the number of potentially eligible participants and the number recruited and excluded due to various reasons. We have now slightly changed the diagram and tried to simplify it as per the suggestion of the reviewer.

**17. What was the mean duration of the follow up?**

- Response: The follow up of the patients was done within 2 weeks of the first visit. The patients visited the clinic as per their convenience and schedules of the pain physician. All the follow ups were either at the 7^th^ day or 14^th^ day depending on their symptoms. However, we did not calculate the mean duration of the follow up.

**18. Could the authors provide any info on other sociodemographic variables such as ethnicity, education, occupation etc. These are important sociodemographic parameters that relates with the external validity and utility of the scale.**

- Response: We have added the ethnicity of the participants in Table 1. We did not look into the other variables like education and occupation since we did not feel much relevance of these variables with neuropathic pain based on our clinical experience.

**19. Table 4. Unclear what the ICC means in this Table. The results on ICC following this (text and Table 5) is quite high compared to what is presented in Table 4. Is it supposed to be pin prick?**

- Response: It is mentioned in the footnote of table 4 what the ICC denotes. Table 4 shows the ICC of internal consistency reliability that measures how well the tool addresses different constructs. On the other hand, Table 5 shows the ICC of the test-retest reliability. Therefore, these values came different. Yes, we have corrected the word pin prick.

**20. Table 6 is an intense table. It should be simiplied so that it is self explanatory.**

- Response: Thank you for the comments. We have tried to make the table relatively simple. We removed the scores of positive and negative likelihood ratios as these were derived out of the sensitivity and specificity values which we have already showed in the table.

**DISCUSSION:**

**see the major criticism comments above.**

**21. Limitations: An important aspect of construct validity, that is hypothesis testing is missing. That is testing strength and direction of association of the scale with other established scales (that is translated, cross-culturally adapted and validated scales) that assess the same or related constructs. This could be a recommendation for the future studies.**

- Response: During our study period, there was no other validated Nepali scale for testing the neuropathic signs and symptoms. The LANSS scale which was validated by Sharma et. al in Nepali was published only in 2022. In future studies, both of these tools can be used for testing the construct validity.

**22. Limitations: Unclear who the study findings can be generalised to, because the full demographics of the study participants are unclear (e.g., sociocultural and economic positioning including education, ethnicity, occupation, income levels)**

- Response: Our study findings can be generalized to all those who can communicate in Nepali, irrespective of their demographics like education, ethnicity, occupation and income. We also mentioned in the discussion that we did not face any difficulty during data collection because of the difference in literacy level.

**CONCLUSIONS:**

**As above, rather than predicting "neuropathic pain" it should be "identifying neuropathic pain signs and symptoms".**

- Response: The word has been revised as per the suggestion.

We feel that the paper is much improved as a result of this peer review process. We are really grateful to the reviewers and editor for helping us take the manuscript to this stage. We hope that the amendments made would meet your expectations. We are completely open to any further suggestions.

Your's sincerely

Sujata Shakya

Corresponding author
