# Supplementary material for: Nepalese version of Douleur Neuropathique 4 (DN4) questionnaire for detection of neuropathic pain signs and symptoms: Translation and psychometric properties
Source: PLoS One. 2023 Jul 17;18(7):e0287737. doi: 10.1371/journal.pone.0287737 (PMC10351728; doi:10.1371/journal.pone.0287737)
Supplement: Attachment — Submitted filename: Response to Reviewers (4).docx [file pone.0287737.s008.docx]

To

Dr. Saurab Sharma

Guest Editor, PLOS ONE

**RE: PONE-D-21-36866 - “Nepalese version of Douleur Neuropathique 4 (DN4) questionnaire for assessment of neuropathic pain: a validation study”**

Dear Dr. Saurab Sharma,

Thank you so much for your email received on 17^th^ November 2022 and the valuable suggestions of the reviewers. First of all, we are grateful to have you as the guest editor of the journal. We have attempted our best to address all these comments and give a point-by-point responses.

Please find attached the following for your kind consideration:

1) A rebuttal letter that responds to each comment of the editor and the reviewer
2) A marked-up copy of the manuscript highlighting changes as per the editor’s and reviewers’ comments.
3) A clean version of the revised manuscript without track changes

Hope the changes made have addressed all your comments and suggestions. We are always available for any further information if required.

Yours Sincerely,

Sujata Shakya

Corresponding Author

**POINT BY POINT RESPONSES TO THE COMMENTS OF THE EDITOR AND REVIEWERS**

**PONE-D-21-36866**

**Response to the editor and reviewer**

- Response: We thank the editor and reviewer for reviewing the manuscript and very useful and encouraging comments. We have revised the manuscript in line with your suggestions. The specific changes and responses to the different points raised are mentioned below. Your comments are highlighted in bold followed by our responses.

**Response to the editor**

**1. The Introduction does not completely cover “what is known” and the need for the study or “what this study adds to what is known” at the time of writing the paper. Consistent with the comments in the previous round of review, the Introduction needs to cover relevant literature on (neuropathic) pain in Nepal. I appreciate you cited Sharma et al (2019) Pain Reports paper to identify gaps in the neuropathic literature. However, a paper published online in September 2021 (Journal of Pain; https://pubmed.ncbi.nlm.nih.gov/34583020/ ), identified the prevalence of neuropathic pain signs and symptoms as 12% in the general population and 23% in people with chronic pain. This is probably the only relevant publication on the topic in Nepal so far. The authors also indicated that the paper was published after they conceived the study, which is a fair point that this study couldn’t influence the conception and design of the current study (and therefore couldn’t be included in the current study). However, it is standard practice is to review relevant literature – at the time of manuscript writing – and include new papers to highlight “what is known”. A publication does not necessarily dismiss the need for another study (or a screening tool in this case). However, the authors should appreciate the literature published at the time of the writing and highlight what gap this current paper adds to what is known. The authors seem to have ignored the advancement in the assessment of neuropathic pain in Nepal (i.e., cross-cultural adaptation of the validation of the SLANSS, https://pubmed.ncbi.nlm.nih.gov/34583020/) in the Introduction and Discussion of the current paper. As I see it from my perspective the validation of DN4 in Nepali is an important step in the assessment of neuropathic pain in clinical populations whereas the SLASS was validated in a community sample with the intent for self-administration in community samples. These two measures complement each other and perhaps there is a nice opportunity to compare these two instruments in future research. For tips around writing clear Introduction in the field of Epidemiology, you may refer to** [**https://pubmed.ncbi.nlm.nih.gov/23497856/**](https://pubmed.ncbi.nlm.nih.gov/23497856/)**.**

- Response: Thank you for the valuable comments and suggestion. As suggested by the Editor of the journal, we have now revised the introduction section and tried to highlight on what is known, and what this study adds in line numbers 94-112. In addition, as suggested, we have explained and cited the article on validation of Nepalese version of S-LANSS in line numbers 104- 106. This was not under our notice since our study was conducted in 2019. Thank you again for pointing on this.

**2. Methods: Line 189, specify what criteria were considered for a probable diagnosis of neuropathic pain. Specify what patient’s history and clinical examination contributed to this decision. As indicated in the previous review, this is critical for the internal validity of the study.**

- Response: The probable diagnosis of neuropathic was totally based on the decision of the physician who examined the patient for the first time and we highlighted this in line numbers 187, 188. The patient’s symptoms and examination in the same neurological distribution were looked for by the physician. However, the researcher could not interfere with the physician’s final decision, and this decision was considered as the standard for comparing the results of DN4 questionnaire. This was also mentioned as the major limitation in the discussion part.

**3. Line 208, I would have thought ICC agreement is suitable rather than “consistency”. Justify the relevance of “consistency” over “agreement”. Similarly, why is it ICC(3,1) and not ICC(2,1)?**

- Response: We heartily thank the editor for the clarification and feedback. After thorough review of literature, we came to know that there should be ICC agreement instead of consistency in case of test-retest reliability. We have corrected this in our manuscript in line number 210. However, since it was a two way mixed effect model (not random effect), and the single rater measurement, we came to the conclusion that the form of ICC should be ICC (3,1) and not ICC(2,1). We have highlighted this in line numbers 210, 211.

**4. Results: Table 4. A follow-up clarification to the previous comment. Authors state that in their response to reviewers that “Table 4 shows the ICC of internal consistency reliability”. However, this ICC is used for test-retest reliability NOT for internal consistency. Cronbach’s alpha is used for internal consistency unless there is other credible evidence for this. Please add such references to justify your choices.**

- Response: Thank you for your suggestion. We are now clear on this and have removed the value of ICC in the analysis of internal consistency reliability and sticked this to the test-retest reliability only in Table 4.

**5. All results related to ICC and Cronbach’s alpha should be reported in decimals (report up to 3 digits after decimals).**

- Response: As mentioned by the editor, we had already reported in our earlier version of the manuscript, the ICC and Cronbach’s alpha in decimals with 3 digits after decimals in Table 4 and Table 5.

**6. The Discussion section still remains suboptimal (also corroborated by Reviewer 3). The authors also ignored the previous comments to adopt an established approach to reporting the Discussion section. In order to meet the requirements for the journal, please adopt PLOS’s recommendation on writing Discussion (see https://plos.org/resource/how-to-write-conclusions/). Lines 291-302 (of the track changed version of the manuscript) are not relevant to the Discussion section and should be deleted. Sections from lines 303 to 319 may be moved to Results but should be abbreviated by retaining “Results” aspects of the texts. This section needs restructuring and writing.**

- Response: We heartily thank the editor for referring to the literature on how to report the discussion section. We have revised our manuscript according to the recommendations provided in the literature. As suggested, lines 291-302 has been deleted and the lines 303-319 were moved to the results section (line numbers 244-254).

**7. Limitations: authors seem to have ignored two of the previous comments related to the study limitations (also identified by Reviewer 3. First, the authors (all reasons considered), did not assess construct validity using hypothesis testing (see COSMIN guidance on its importance). This should be acknowledged as a limitation and could be promoted as a recommendation for future studies on DN4.**

- Response: Thank you for the suggestions. During our study, there were no other standard Nepalese tools to compare and assess the construct validity. We have mentioned this in our limitations (line numbers 394-395). However, with the availability of two different tools, we have provided recommendation to compare two tools for the future studies (line numbers 405-407).

**8. The authors did not assess education as their study variable but stated that both literate and illiterate patients were included in the study (lines 352-353). We cannot comment on something that wasn’t measured. Also in the previous response, the authors stated “Our study findings can be generalized to all those who can communicate in Nepali, irrespective of their demographics like education, ethnicity, occupation and income. We also mentioned in the discussion that we did not face any difficulty during data collection because of the difference in literacy level.” This is not true. If this was true, Nepali translation of DN4 wouldn’t be required provided it is shown to be valid in multiple other languages and socioeconomic groups. The study is only generalisable to the population on which they are tested. If the sample consisted of all participants with high levels of education, the study can’t be generalised to those who can’t read or write. Social determinants of health are important in research and health care, also endorsed by the World Health Organisation (https://www.who.int/health-topics/social-determinants-of-health#tab=tab_1). See more on social determinants of health here (https://pubmed.ncbi.nlm.nih.gov/24189091/ and https://www.ncbi.nlm.nih.gov/pmc/articles/PMC3863696/). Not assessing socioeconomic factors is an important limitation and future studies should consider assessing these.**

- Response: Thank you for the valuable comments and suggestion. We accept that we did not consider the education as our study variable, which can influence the result. This is our limitation which we have mentioned in line numbers 392-394). However, we did not mean to ignore the importance of social determinants of health as endorsed by the World Health Organisation.

**Response to the reviewer**

**Abstract:**

**“This study aimed to translate and validate DN4 questionnaire into Nepalese version.”**

**The use of this phrase implies that “validate” is something you do to a test and that “validity” is a property of the test itself.**

**“Tests do not have reliabilities and validities, only test responses do. This is an important point because test responses are a function not only of the items, tasks, or stimulus conditions but of the persons responding and the context of measurement.” SEE: Messick S. Validity. In: Linn RL, editor. Educational Measurement. 3rd ed. Phoenix: ORYZ Press; 1993. p. 14.**

**Validity processes are aimed not at the integrity of the measures themselves, but about inferences that can be made about the people who complete those measures.**

**I would suggest that the term “validate the measure (DN4 questionnaire)” be changed throughout the entire paper. For example, you may recast as: “This study aimed to translate the DN4 questionnaire into Nepalese and assess its psychometric properties.”**

- Response: We have found in different literature using varied terms like validation in some and assessment of psychometric properties in others. Therefore, we had used the term validation in our study. However, we totally agree with the reviewer in using the terms ‘psychometric properties’ and thus changed our title, objective and other sections accordingly throughout the manuscript excluding while reporting the articles where the term ‘validation’ has been used.

**Method:**

**1. Did you obtain permission to translate the DN4 questionnaire?**

- Response: Yes, we obtained permission to translate the DN4 questionnaire by email. The principal author of the paper on original version of the DN4 questionnaire granted permission for translation in condition that our study is not sponsored by any private company. We have mentioned this in our manuscript in lines 148-149.

**2. How did the authors handled missing data?**

- Response: There were no any missing data in our study, and we reported this in the results section of our manuscript in line number 243.

**3. As far as the methodology is concern, no any form of validity test (i.e. content, structural and criterion validity) has been conducted even though the authors used the COSMIN checklist.**

**With the study sample size, the authors could have at least assess structural validity of the Nepalese DN4 questionnaire**

- Response: We thank the reviewer for this feedback. Actually, as we used the physician’s diagnosis as the gold standard to assess how good our instrument score was for classification purpose. So, we obtained the Area under the curve which showed that our test was strong. In the revised manuscript, we have now evaluated the Kappa statistics to analyze the agreement between the findings of DN4 questionnaire and the gold standard (physician’s diagnosis), which showed substantial agreement. We have also evaluated sensitivity and specificity with a preexisting cutoff. We totally agree that we could have assessed structural and other validity methods, and we have plan to expand on this in our future studies.

**4. “The Area under the Curve (AUC) was considered as the area between the two episodes of the tests” What do you mean by two episodes? Are you referring to test and retest? If so, it sounds more appropriate to use the latter.**

- Response: Thank you for your suggestion. We have reworded the terms ‘episodes’ into ‘test and retest’ throughout the manuscript.

**5.**

**Discussion:**

**I would suggest to the authors to rearrange the flow of the discussion as follows:**

**a. Translation and cultural adaptation**

**b. General characteristics including response of the questionnaire**

**c. Responsiveness (sensitivity to change)**

**d. Reliability**

**e. Strengths and limitations**

**f. Implication to clinical practice and research**

- Response: We are very thankful for this wonderful suggestion. We have rearranged our discussion section into the above mentioned subheadings, after which we felt that it helped a lot to arrange systematically. We have also added description on the implication of our study in line numbers 397-402.

We feel that the paper is much improved as a result of this peer review process. We are really grateful to the reviewers and editor for helping us take the manuscript to this stage. We hope that the amendments made would meet your expectations. We are completely open to any further suggestions.

Your's sincerely

Sujata Shakya

Corresponding author
