# Supplementary material for: Nepalese version of Douleur Neuropathique 4 (DN4) questionnaire for detection of neuropathic pain signs and symptoms: Translation and psychometric properties
Source: PLoS One. 2023 Jul 17;18(7):e0287737. doi: 10.1371/journal.pone.0287737 (PMC10351728; doi:10.1371/journal.pone.0287737)
Supplement: Attachment — Submitted filename: Response to Reviewers.docx [file pone.0287737.s009.docx]

To

Dr. Saurab Sharma

Guest Editor, PLOS ONE

**RE: PONE-D-21-36866R2 - “Nepalese version of Douleur Neuropathique 4 (DN4) questionnaire for detection of neuropathic pain signs and symptoms: translation and assessment of psychometric properties”**

Dear Dr. Saurab Sharma,

Thank you so much for your email received on 23^rd^ February 2023 and the valuable suggestions. We have attempted our best to address all the comments and give a point-by-point responses.

Please find attached the following for your kind consideration:

1) A rebuttal letter that responds to each comment of the editor
2) A marked-up copy of the manuscript highlighting changes as per the editor’s comments.
3) A clean version of the revised manuscript without track changes

Hope the changes made have addressed all your comments and suggestions. We are always available for any further information if required.

Yours Sincerely,

Sujata Shakya

Corresponding Author

**POINT BY POINT RESPONSES TO THE COMMENTS OF THE EDITOR**

**PONE-D-21-36866R2**

**Response to the editor**

- Response: We thank the editor for reviewing the manuscript and very useful and encouraging comments. We have revised the manuscript in line with your suggestions. We have also done language corrections by consulting with the English language expert. The specific changes and responses to the different points raised are mentioned below. We have specified the respective line numbers based on the track changed version of the revised manuscript. Your comments are highlighted in bold followed by our responses.

**Methods:**

**1. Report completely the scoring of DN4 (that is 0 to 10) and “presence of neuropathic pain signs and symptoms” and “absence of neuropathic signs and symptoms” using a cut score of 4 if this is true. You also write later that cut off 4 yielded the highest sensitivity and specificity, I am unsure where that interpretation came from.**

- **Response**: Thank you for your suggestions. In our revised manuscript track change version line number 244-246, we explained about using cut-off score of 4 for dichotomous categories of Pain assessment findings. We have now deleted ROC curve and all interpretations on this based on the earlier online video conversation. We also removed the words ‘cut-off 4 yielded the highest sensitivity and specificity’ of line numbers 395, 396, 465, 466.

**2. Explicitly say, which of these scorings were used for specific data analyses. Example, dichotomous scoring of “yes” or “no” for neuropathic pain for Kappa and 0 to 10 score for ICC (note both of these are reliability test).**

- **Response**: We have now clearly indicated the scoring that we used for ICC and kappa in line numbers 274, 275 of track changed version. We have included the kappa coefficient for assessing reliability test in table 6 and removed from table 3.

**3. Be specific with your statements. You write “Under descriptive statistics, mean, frequency and percentage were calculated.” Mean for which outcome or variable? Frequency for which? Etc….**

- **Response**: Thank you for your suggestions. We have revised and now mentioned the specific descriptive statistics for the respective variables in line numbers 238-241 of track changed version.

**4. Cohen’s Kappa is used for test-retest reliability for dichotomous outcomes. You seem to have used it for diagnostic accuracy. See your text, “To compare the agreement between the score of DN4 questionnaire and the physician’s diagnosis of pain, Cohen’s kappa statistics was used”. This is incorrect but you are welcome to provide a rebuttal. If so, cite a credible reference to support this.**

- **Response**: Thank you so much for your comments. We now realized that we made a mistake by using Cohen’s kappa for diagnostic accuracy. It should be used for test retest reliability. Now we have included Cohen’s kappa statistics for primarily calculating test retest reliability (table 6) and then ICC as the secondary analysis (S3_Table). We have explained this in our methods section in line numbers 274, 275.

**5. In response to my editor’s comment in the previous round, you declined the suggestion of ICC2,1 (2 way random model) and retained the use of ICC3,1 (2 way mixed model) without any convincing explanation. Your choice of ICC3,1 means that your analyses will not be generalisable to other clinicians using DN4 but is only valid for the clinicians in the study. This contradicts with your conclusion that “…it can now be used as a screening tool for ….” For more reading** [**https://www.ncbi.nlm.nih.gov/pmc/articles/PMC4913118/**](https://www.ncbi.nlm.nih.gov/pmc/articles/PMC4913118/)**.**

- **Response**: We have now changed ICC from ICC(3,1) to ICC(2,1) in line numbers 279-280 since we agreed that using ICC(2,1) indicates generalizability of the study findings to other clinicians and researchers.

**6. ROC: You say “We then derived Receiver Operating Characteristic (ROC) Curve for analyzing the discriminative ability of the test. The ROC curve was plotted for two episodes of the test, with sensitivity versus 1-specificity over all possible cutpoints.” Discriminatory ability of which test and against which test? Could you clarify what you mean by “two episodes of the test”.**

- **Response**: As per the suggestion during online conversation, we removed ROC curve from our data analysis and results and only included the 2X2 contingency table to compare the index test and reference test (table 3).

**7. What type of scoring of DN4 was used for ROC curve (dichotomous versus 0-10)?**

- **Response**: We have now deleted the ROC curve from our data analysis.

**8. You randomly introduce the use of Responsiveness as a title for a paragraph in Discussion. The study did not aim to assess responsiveness and is not applicable to this study.**

- **Response**: We are really grateful to the editor for pointing out this error. We removed the word ‘responsiveness’ from the discussion section and changed it to diagnostic accuracy in line number 455.

**9. “Similarly, different diagnostic values to detect neuropathic pain like sensitivity, specificity, positive and negative predictive value, and positive and negative likelihood ratio were calculated.” – Say how?**

- **Response**: Thank you for your suggestions. In the revised version, we described specifically in the methods section how each of the above measures were calculated in our study. These were explained in line numbers 249-265.

**10. Use of 2x2 contingency table would be useful for the readers with diagnosis of neuropathic pain signs and symptoms by the index test (using DN4’s dichotomous categories) and reference test (doctor’s diagnosis).**

- **Response**: We included 2X2 contingency table highlighting the association between the diagnosis of neuropathic pain signs and symptoms by the index test and the reference test (table 3).

**11. You use PPV and NPV without spelling them out for the first time in the main text.**

- **Response**: We thank the editor for this comment. We have now done corrections accordingly in line numbers 257-259.

**Results:**

**12. Please upload PDF version of the Nepali DN4 instead of TIFF version. Make sure to refer ALL the Supplementary Tables and Figures in the Main text .**

- **Response**: We have uploaded the PDF version of the Nepali DN4 tool as suggested. We also included short description of the supplementary tables and figures in the main text of the manuscript.

**13. Table 2 reports comparison of frequency of items of DN4. But statistical tests used to for this is not presented in Methods section (name of the test in table legend does not suffice). Nor this was an aim of the study. Please list the complete aims of the study. In the same order, present the data analysis methods. Likewise, use the same order to present your results and discuss it. This logical order makes it easier for the readers to read and understand the findings. It is also easier for authors to write in this structure.**

- **Response**: Thank you so much for your suggestion. We have removed the statistical test in table 2 and now presented only the frequency table of different items of DN4 questionnaire in neuropathic and non-neuropathic pain signs and symptoms (table 2). We have included this in our methods section as well in line numbers 240, 241. As suggested, we have tried our best to include all the explanations in each section of the manuscript in the same logical order.

**14. Tables 2 and 3 uses the diagnosis of pain as “nociceptive” for participants who were not categorised as having “neuropathic”. Not-being neuropathic does not automatically qualify as “nociceptive”. I recommend authors to use “non-neuropathic” instead. Please make sure to use this consistently and throughout the manuscript (like you use in Table 5).**

- **Response**: We totally agree with the editor’s comments and now changed the word from ‘nociceptive’ to ‘non-neuropathic’ in tables 2 and 3 and throughout the manuscript.

**15. Delete the use of “reliability” after “internal consistency” throughout the paper.**

- **Response**: As per the suggestion, we have deleted the word ‘reliability’ throughout and just included the words ‘internal consistency’.

**16. Line 348: use “Diagnostic accuracy of DN4” instead of ROC curve analysis as the heading.**

- **Response**: We have now deleted the ROC curve from our analysis.

**17. Table 4, use the words and phrases exactly as they appear in the original DN4 questionnaire for ALL items. Eg Painful cold instead of cold pain, Pins and needles instead of Needle sensation. These may mean different things.**

- **Response**: Thank you for your valuable comments. We totally agree that the words should exactly be same as in the original questionnaire. We have kept the words and phrases that were used in the original DN4 questionnaire throughout the manuscript and also in table 2 and table 5.

**18. On line 353, Fig 1 is referred but it isn’t located in the main text. All figures referred to as Figures should be presented in the main text rather than in online supplement.**

- **Response**: We have removed figure 1 and now made sure that each table is presented in the main text and not in the online supplement.

**19. When you say the best sensitivity and specificity scores were identified for a cut off of 4. How was this determined? There is no information on the manuscript around this.**

- **Response**: We apologize for this error. Now we have deleted the sentences of line numbers 395, 396, 465, 466.

**20. Line 355, “Similarly, other measures like Youden index, positive and negative predictive values were also calculated (Table 6).” This info is relevant for Methods section with more details on how to interpret them. There is no mention of Youden index in data analyses section.**

- **Response**: Thank you for your comments. We have now described the use of Youden index and its explanation in the methods section in line numbers 254-257.

**Discussion:**

**21. In paragraph 1, summarise the key results instead of just listing the aims and data analysis plan.**

- **Response**: The summary of key results has been added in line numbers 418-423.

**22. “DN4 questionnaire was first created by French neuropathic team and was originally in French language. Due to its simplistic nature, the questionnaire has been translated into various other languages. However, translation can be a complicated process. The major hurdle is the fact that every language has its own unique nuances, grammatical rules, vocabulary, and so on. Rendering ideas from one language to another while also keeping the essence intact is a difficult task with a simple translation” is background information and is not relevant for Discussion. Please remove it. Instead only discuss the results of the current study.**

- **Response**: The whole paragraph has been removed of line numbers 427-432.

**23. Line 399: “In the Hindi version of DN4 questionnaire also, cold pain was present in only 14% of patients with neuropathic pain [9]. In our study, only 17 % of patients with neuropathic pain had symptoms of cold pain.” This info does not fit under the heading of “translation…”. Only include issues related to translation here.**

- **Response**: This part has been removed from heading of translation of line numbers 442-444.

**24. In this paragraph, you mention about “cold pain” only. How about other items on the scale? It was help readers with this info for their clinical practice. In our own previous study to identify how people describe their chronic pain, we found a lot of interesting words and phrases to describe pain by Nepali people with chronic pain (https://pubmed.ncbi.nlm.nih.gov/27895511/). If there were no problems with other items, please say so.**

- **Response**: We have added discussion about the different words use by Nepalese to describe electric pain in line numbers 432-438. Earlier we did not include this in the discussion but later we realize that the readers will also know about different words to describe the same item. Thank you for suggesting.

**25. Line 439: as above, unsure how you concluded that 4/10 was the best cut off point. In fact this wasn’t the main your aim nor you tested this. I recommend you delete this throughout.**

- **Response**: We have now deleted that 4/10 is best cut off point in our study of line numbers 453, 454**.**

**26. As above, replace responsiveness heading (line 441) with Diagnostic accuracy. Also, Kappa isn’t a measure for diagnostic accuracy but is for reliability. Your analyses should be revised and correct both for reliability and diagnostic accuracy.**

- **Response**: We have revised this mistake and now replaced the heading responsiveness with diagnostic accuracy in line number 455. We included in the discussion section under the heading reliability that kappa was used for test-retest reliability in line number 478.

**27. Line 465 and 466, in the previous round of review, I pointed out that ICC values should be in decimal places, and your response was that this was corrected in all locations although it was’t. As an editor, it is frustrating when authors say the errors are corrected when they are not. It can be perceived as attempt to deceive.**

- **Response**: We are really sorry for this mistake. We failed to correct this mistake in discussion part in our earlier revision. Now, it has been corrected in line numbers 477, 478.

**28. Strengths and limitations: unsure how interviews minimize reliability and validity? Interviews in fact can lead to socially desirable response and interviewers can influence the responses affecting the validity of the responses. Also, DN4 has components that need clinical assessment unlike self-reported measures. So, this discussion point is irrelevant, and I recommend deleting.**

- **Response**: This line has been deleted (line numbers 486-488).

**29. Limitation 1: “The patients were first seen by a pain physician who was not involved in the interview.” This isn’t a limitation really but is in fact a strength. Ideally, the index test and the reference test should be conducted independently maintaining blinding by separate clinicians. This blinding ensures the robustness of the diagnostic accuracy tests. See QUADAS-2 tool for more details √ (https://pubmed.ncbi.nlm.nih.gov/22007046/). I also recommend using the terms such as index test and reference test to describe the tests as subheadings in Methods section.**

- **Response**: We have included this under strength in line numbers 488, 490. We have used index test and reference test in Methods section (line numbers 219-221, 227, 230) and throughout the manuscript.

**30. The limitation here should be, in line with my comments in the previous round of review, “reference test for diagnosis of neuropathic signs and symptoms was performed by a pain physician using clinical examination alone” or something along this line.**

- **Response**: We highlighted that our study limitation was that the reference test for diagnosis was based on history taking and clinical examination (line numbers 492-497).

**31. Implication: while it is true that DN4 could be used in epidemiological studies, but it will demand enormous resources as it will require trained clinicians to perform physical examination increasing the costs of the study significantly as opposed to using self-reported tools alone. This further contradicts with authors’ point of its utility in “developing countries” which are resource limited. I would recommend authors to align the discussion around this or focus the implications around the clinical utility of DN4 rather than on epidemiological studies. Relevant to this, please change the use of “epidemiological” from conclusions of your abstract to “research settings”**

- **Response**: The DN4 questionnaire required physical examination which can be performed only by trained medical personnel, so we realized that this DN4 questionnaire is relevant in research setting so the word epidemiological has been removed throughout (line numbers 69, 103, 108, 509) and the implication has been highlighted towards the clinical utility of the questionnaire in line numbers 511, 512. Thanks for this analysis.

**Other comments:**

**32. A general comment: as DN4 is a screening or diagnostic tool, test-retest reliability isn’t the primary concern here. Instead the focus should have been on inter- or intra-rater reliability. Test-retest reliability is critical for tests that are prognostic or that are used as outcome measures to track change within group or difference between groups.**

- **Response**: We admit that the best approach would have been using inter-rater reliability. However, we could not perform it due to difficult clinical settings of our hospital.

**33. Title: I recommend adding detection of neuropathic pain “signs and symptoms” in order to factor in its limitation of diagnostic accuracy in line with my feedback in the previous round of the review.**

- **Response**: The neuropathic sign and symptoms has been added throughout the manuscript.

**34. Abstract: Needs to be significantly revised in order to give it a structure that I was referring in above. Aims should include all aspects of measurement properties: internal consistency, test-retest reliability, diagnostic accuracy. In Methods, write how these were assessed in the same order. Results: report the results also in the exact same order. Unclear what “strength of test” means in line 45. It is confusing to read about AUC without any mention in Methods. Also, there was no mention of first and second test in the Methods.**

- **Response**: The study aim in the abstract has been revised accordingly (line numbers 32, 33) and we now revised the order of reporting the measures and results in all sections of the abstract.

We are really grateful to the editor for helping us take the manuscript to this stage. We hope that the amendments made would meet your expectations. We are completely open to any further suggestions.

Your's sincerely

Sujata Shakya

Corresponding author
